# Effects of sub-lethal high-pressure homogenization treatment on the outermost cellular structures and the volatile-molecule profiles of two strains of probiotic lactobacilli

**DOI:** 10.3389/fmicb.2015.01006

**Published:** 2015-09-23

**Authors:** Giulia Tabanelli, Pamela Vernocchi, Francesca Patrignani, Federica Del Chierico, Lorenza Putignani, Gabriel Vinderola, Jorge A. Reinheimer, Fausto Gardini, Rosalba Lanciotti

**Affiliations:** ^1^Centro Interdipartimentale di Ricerca Industriale Agroalimentare, Università degli Studi di BolognaCesena, Italy; ^2^Unit of Metagenomics, Bambino Gesù Children's Hospital, IRCCSRome, Italy; ^3^Dipartimento di Scienze e Tecnologie Agro-alimentari, Università degli Studi di Bologna – Sede di CesenaCesena, Italy; ^4^Unit of Parasitology, Bambino Gesù Children's Hospital, IRCCSRome, Italy; ^5^Facultad de Ingeniería Química, Instituto de Lactología Industrial (INLAIN, UNL-CONICET), Universidad Nacional del LitoralSanta Fe, Argentina

**Keywords:** high-pressure homogenization, probiotic lactobacilli, MALDI-TOF MS, transmission electron microscopy, buttermilk, volatile profile

## Abstract

Applying sub-lethal levels of high-pressure homogenization (HPH) to lactic acid bacteria has been proposed as a method of enhancing some of their functional properties. Because the principal targets of HPH are the cell-surface structures, the aim of this study was to examine the effect of sub-lethal HPH treatment on the outermost cellular structures and the proteomic profiles of two known probiotic bacterial strains. Moreover, the effect of HPH treatment on the metabolism of probiotic cells within a dairy product during its refrigerated storage was investigated using SPME-GC-MS. Transmission electron microscopy was used to examine the microstructural changes in the outermost cellular structures due to HPH treatment. These alterations may be involved in the changes in some of the technological and functional properties of the strains that were observed after pressure treatment. Moreover, the proteomic profiles of the probiotic strains treated with HPH and incubated at 37°C for various periods showed different peptide patterns compared with those of the untreated cells. In addition, there were differences in the peaks that were observed in the low-mass spectral region (2000–3000 Da) of the spectral profiles of the control and treated samples. Due to pressure treatment, the volatile-molecule profiles of buttermilk inoculated with treated or control cells and stored at 4°C for 30 days exhibited overall changes in the aroma profile and in the production of molecules that improved its sensory profile, although the two different species imparted specific fingerprints to the product. The results of this study will contribute to understanding the changes that occur in the outermost cellular structures and the metabolism of LAB in response to HPH treatment. The findings of this investigation may contribute to elucidating the relationships between these changes and the alterations of the technological and functional properties of LAB induced by pressure treatment.

## Introduction

Recently, there has been increasing interest in the potential of applying techniques such as pulsed electric field (PEF), high-hydrostatic pressure (HHP), or high-pressure homogenization (HPH) to enhance the survival rate of probiotic strains or to modify their overall functionality in a positive manner. Among these processes, HPH has been proposed for the treatment of raw materials or the sub-lethal treatment of starters or non-starters and probiotic cells for use in the production of probiotic fermented milks or cheeses with improved sensorial, technological, or functional properties (Lanciotti et al., 2006; Burns et al., 2008a; Patrignani et al., 2009; Tabanelli et al., 2012).

Lanciotti et al. (2007a) demonstrated that a sub-lethal HPH treatment could control the fermentation kinetics of bacterial strains used as starters and modify their metabolic profiles, leading to products with enhanced sensorial properties. Moreover, although the responses varied according to the characteristics of individual strains of lactic acid bacteria (LAB), HPH increased the activity of extracellular or cell wall-associated proteolytic enzymes without having detrimental effects on their viability, confirming their tolerance of moderate pressures.

In addition, sub-lethal HPH treatment improved the acid tolerance and bile tolerance of *L. acidophilus* LA-K (Muramalla and Aryana, 2011) and enhanced some of the biological and functional properties of known probiotic strains both *in vitro* and *in vivo*, and more specifically in mice, trials (Tabanelli et al., 2013, 2014). In particular, the latter authors demonstrated that an HPH treatment applied at 50 MPa modulated the hydrophobicity and auto-aggregation of the treated strains *in vitro* and modified their interaction with the small-intestinal structures of BALB mice. The HPH-treated cells showed a different behavior in the mouse gut and induced a stronger IgA response compared to those of untreated cells, in strain- and feeding-period-dependent manners. These effects were attributed to HPH having modified the outermost cellular structures that play roles in the interactions of probiotic cells and immune cells and are the main targets of sub-lethal pressure (Muramalla and Aryana, 2011; Tabanelli et al., 2014). It is known that when pressure is applied at a sub-lethal level, various cellular responses occur and that the composition of the cellular membrane can change to withstand the exposure to a sub-lethal stress (Russell et al., 1995). Tabanelli et al. (2014) showed that the composition of the membranes and their unsaturation levels affected the response mechanisms adopted by probiotic strains, such as *L. paracasei* A13 and *L. acidophilus* DRU, when they were subjected to sub-lethal HPH treatments. In particular, these authors reported that these treatments reduced the level of cyclic fatty acids and increased the unsaturation level, leading to modification of the membrane fatty-acid profile in a strain-dependent manner. Some authors showed that modulating the membrane fatty-acid composition in response to environmental conditions affected the cell-surface hydrophobicity and adhesive ability of bacterial strains and, consequently, their functional features (Kirjavainen et al., 1998; Kankaanpää et al., 2001, 2004).

Considering that some probiotic properties are associated with the bacterial cell wall, which is also the principal target of HPH, the aim of this study was to evaluate the effect of sub-lethal HPH treatment on the outermost structures of two strains endowed with probiotic features (*Lactobacillus acidophilus* DRU and *Lactobacillus paracasei* A13) using transmission electron microscopy (TEM). These strains were chosen based on the results of previous studies that demonstrated their ability to increase certain functional properties in response to HPH treatment (Tabanelli et al., 2012, 2013). Moreover, bacterial profiling analyses of treated and untreated strains were performed using MALDI-TOF MS (matrix-assisted laser desorption ionization time-of-flight mass spectrometry) to evaluate the effect of HPH on the cellular peptide profiles. MALDI-TOF MS has been applied to microbial detection because it generates characteristic mass spectra that are unique for each species, permitting identification at the genus and species levels, and potentially, at the strain level (Croxatto et al., 2012). This technique has also been employed to evaluate changes in the peptide profiles of microbial cells that were induced by various growth conditions and physico-chemical stresses (Šedo et al., 2013). The high level of versatility and the speed and accuracy of this methodology played key roles in its adoptation in many fields, including clinical diagnostics, environmental monitoring, and food-quality control. Although MALDI-TOF MS analysis is an interesting approach to microbial characterization, it has rarely been applied to food-related microorganisms.

Finally, the effect of HPH treatment on the metabolism of LAB in a dairy product, such as buttermilk, was investigated; buttermilk was chosen as the vehicle for the probiotic cells because it was reported to be a suitable medium for maintaining adequate levels of LAB during refrigerated storage (Burns et al., 2008b). Additionally, the volatile profiles of buttermilk inoculated with treated or control cells during 30 days of refrigerated storage were investigated to evaluate the impact of the sub-lethal HPH treatment on the accumulation of molecules that can impart aromas to the product.

## Materials and methods

### Strain culture conditions and microbiological analyses

*L. paracasei* A13 and *L. acidophilus* DRU are two commercially available probiotic strains that are commonly used in commercial dairy products (Vinderola et al., 2000). The stock cultures were maintained in de Man, Rogosa and Sharpe (MRS) broth (Biokar, Beauvais, France) containing sterile glycerol (20% v/v) at −70°C in the collection of the Instituto de Lactologia Industrial (INLAIN, UNL-CONICET, Santa Fe, Argentina). Fresh cultures of each strain were obtained by two consecutive passages of a 1% (v/v) inoculum of the frozen stocks in MRS broth, with incubation at 37°C for 18 h under aerobic conditions.

The cell counts were performed before and immediately after HPH. Cell counts were obtained by plating the cultures on MRS agar (37°C, 48 h, under aerobic conditions).

### High-pressure homogenization treatment

The cells in overnight cultures were harvested by centrifugation (8000 g, 10 min, 4°C). The pellets were washed twice using a solution of 9 g NaCl/l and they were re-suspended in sterile phosphate-buffered saline (PBS) (pH 7.4) at a final concentration of approximately 8 log CFU/ml. The cells were subjected to high-pressure homogenization (HPH) at 50 MPa using a PANDA high-pressure homogenizer (Niro Soavi, Parma, Italy). The inlet temperature of the samples was 20°C and the temperature was increased during the treatment at a rate of 3°C/10 MPa. To prepare control samples, the suspended cells were homogenized at 0.1 MPa. Immediately after the treatment, the samples were rapidly cooled to 10°C in a water bath.

### Transmission electron microscopy (TEM)

Transmission electron microscopy (TEM) was used to investigate the morphological changes caused by the HPH treatment. Ten milliliters of the control samples and the HPH-treated samples were centrifuged (8000 g, 10 min) and the pelleted cells were fixed by suspending them in 2.5% glutaraldehyde (in 0.1 M K_2_HPO_4_/KH_2_PO_4_ buffer, pH 7). These samples were stored at 4°C for 2 h. After aldehyde fixation, the samples were prepared according to Bury et al. (2001). The post-fixed cells were washed using the same buffer and then they were dehydrated for 15 min using the following series of ethanol solutions: 50, 75, 90, and 100%. The dehydrated cells were infiltrated with increasing concentrations of Spurr resin (Agar Scientific, Stansted, Essex, United Kingdom) over 24 h. Polymerization of the resin was achieved by heating the samples in an oven at 65°C for 18 h. Thin sections (approximately 90 nm thick) were placed on carbon-coated Formvar-covered 300-mesh copper grids for approximately 15 min, rinsed using 20 drops of distilled water, negatively stained using 6–7 drops of 2% aqueous uranyl acetate and then examined using a Philips CM 10 transmission electron microscope.

### Whole cell MALDI-TOF MS fingerprinting profiles

After being subjected to HPH treatment at 50 MPa, the suspended cells were incubated as follows: (*i*) no incubation (50 MPa T0); (*ii*) incubation at 37°C for 30 min (50 MPa T30); (*iii*) incubation at 37°C for 60 min (50 MPa T60); (*iv*) incubation at 37°C for 120 min (50 MPa T120). To prepare control samples, suspended cells were treated using the homogenizer at 0.1 MPa (0.1 MPa C).

After HPH treatment and incubation, the cells were collected by centrifugation and stored at −80°C until analysis by MALDI-TOF. Then, the cells were washed using H_2_O/CH_3_CH_2_OH (300/600 μl) and were treated according to the method of Putignani et al. (2011). The dried pellets were thoroughly mixed with 50 μl of 70% formic acid (HCOOH) and then with 50 μl of ACN (Sigma-Aldrich, Milan, Italy), and the mixtures were maintained at RT for 10 min at each step. The peptide mixtures derived from acidic hydrolysis were decanted rather than being separated from the insoluble material by centrifugation to avoid possible peptide co-precipitation. These samples were placed (1.5 μl) on an MSP 96 polished steel target (Bruker Daltonics GmbH, Bremen, Germany) and were overlaid with CHCA matrix in 50% ACN/2.5% TFA (1.5 μl) (Sigma-Aldrich) (Putignani et al., 2011). Peptide mass spectra were acquired using a Microflex MALDI-TOF-MS (Bruker Daltonik GmbH) mass spectrometer that was operated in the linear positive mode at the maximum frequency (20 Hz). Spectral measurements were performed using a Microflex LT mass spectrometer (Bruker Daltonics GmbH), using FlexControl software (version 3.0, Bruker Daltonics GmbH). Eight replicates of each spectrum were collected for each species and were analyzed to evaluate the reproducibility of the results, and 500 laser shots/spots were manually collected using the FlexControl software package. The spectral profiles were visualized using FlexAnalysis 3.0 software (Bruker Daltonics GmbH).

### Evaluation of the effects of HPH treatment and the medium pH value on the volatile profiles of buttermilk

To study the effect of HPH treatment on the aroma-compound production of *L. acidophilus* DRU and *L. paracasei* A13 in a dairy medium, the cells were grown in MRS medium for 18 h at 37°C, harvested by centrifugation (8000 g, 10 min, 4°C) and then re-suspended in buttermilk, previously acidified or not to pH 4.6 using lactic acid (Sigma, Milan, Italy), at a level of approximately 8 log CFU/ml. These preparations were HPH treated as described above (paragraph 2.2) at 0.1 MPa (control samples) or at 50 MPa and were stored for 30 days at 4°C. The buttermilk used was reconstituted (77 g/l) from lyophilized buttermilk purchased from a local dairy and was sterilized at 115°C for 30 min. Buttermilk was chosen as the dairy medium for this study because previous studies had shown that it supported the growth and the survival of adequate numbers of LAB cells during storage (Burns et al., 2008b).

The strain viability rate and the aroma profiles of the LAB-containing buttermilk were determined immediately after inoculation and at 15 and 30 days of refrigerated storage. Solid-phase microextraction and gas-chromatography-mass spectrometry (SPME-GC-MS) were used to detect the aroma compounds as reported by Patrignani et al. (2008). Samples (5 g) were placed in 10 ml sterilized vials, sealed using PTFE/silicon septa and heated for 10 min at 45°C, after which the volatile compounds were allowed to adsorb to a fused silica fiber covered with a 75 μm carboxen polydimethylsiloxane (CAR/PDMS StableFlex) (Supelco, Steiheim, Germany). The adsorbed molecules were desorbed in the gas chromatograph for 10 min. The peaks were detected using an Agilent Hewlett-Packard 6890 GC gas chromatograph equipped with a 5970 MSD MS detector (Hewlett-Packard, Geneva, Switzerland) and a Varian Chrompack CP Wax 52 CB capillary column (50 m × 320 μm × 1.2 μm) (Chrompack, Middelburg, The Netherlands) as the stationary phase. The conditions used were as follows: injection temperature, 250°C; detector temperature, 250°C; carrier gas (He); and flow rate, 1 ml/min. The oven-temperature program used was as follows: 50°C for 1 min; increasing from 50°C to 100°C at 2°C/min; increasing from 100°C to 200°C at 6.5°C/min, and then holding at 200°C for 5 min. Volatile-peak identification was conducted via computerized matching of the mass spectral data with those for the compounds contained in the Agilent Hewlett-Packard NIST 98 and Wiley vers. six mass spectral databases. The SPME-GC-MS results for each sample at each time point (un-inoculated and untreated buttermilk at 0, 15, and 30 days of storage) were expressed as the mean values of six independent analyses.

### Statistical analysis of the data

The results of volatile profile analysis of each sample were expressed as the mean values of six independent replicate analyses (conducted on different days) and the data were analyzed using Principal Component Analysis (PCA) using Statistica 6.1 software (StatSoft Italy srl, Vigonza, Italy). Eight replicate MALDI TOF MS Biotyper analyses of each sample were conducted and each replica was considered independently. Prior to performing principal component analysis (PCA)-based hierarchical clustering, each spectrum was subjected to mass adjustment, smoothing, baseline subtraction, Normalization and peak picking. The dendrogram for a single organism was created using with distance measurements (correlation), linkage (average) and a 300-score threshold values using MALDI Biotyper 3.1 software (Bruker Daltonics GmbH). The Pearson's correlation coefficients were calculated using spectral row data using R-Bioconductor to establish the reproducibility of the intra- (replicates) and inter-strain conditional data.

## Results

### Cell viability following HPH treatment

The sub-lethal HPH treatment of *L. paracasei* A13 and *L. acidophilus* DRU cells did not significantly affect their viability, as observed in other studies of LAB strains (Lanciotti et al., 2007a; Tabanelli et al., 2013). The viability rate of the cells of both strains that were HPH treated at 50 MPa was 0.3 log CFU/ml lower than that of the untreated cells.

### Transmission electron microscopy (TEM)

**Figure 1:**
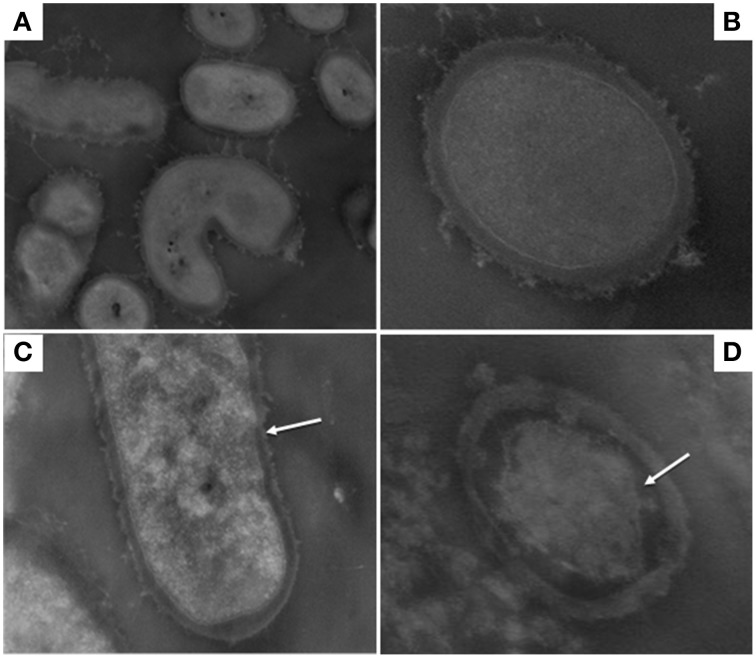
**Transmission electron micrographs of ***Lactobacillus paracasei*** A13: control cells (0.1 MPa) (A,B); 50 MPa HPH treated cells (C,D)**. Magnification: 28,500x **(A)** 73,000x **(B)** and 52,000x **(C,D)**.

**Figure 2:**
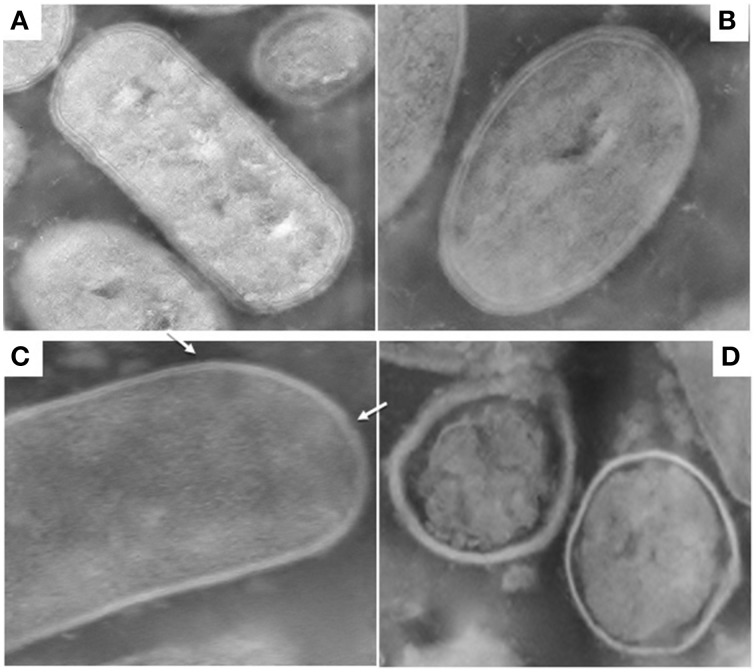
**Transmission electron micrographs of ***Lactobacillus acidophilus*** DRU: control cells (0.1 MPa) (A,B); 50 MPa HPH treated cells (C,D)**. Magnification: 52,000x **(A–C)** and 39,000x **(D)**.

Figures 1, 2 show TEM images of treated and untreated *L. paracasei* A13 and *L. acidophilus* DRU cells. As shown in Figures 1A,B, cell-wall and inner-membrane structures were clearly visible in the control (0.1 MPa) cells of *L. paracasei* A13, as it was an external capsule of proteinaceous material surrounding the cell wall. TEM images of cells of the same strain after HPH treatment at 50 MPa (Figures 1C,D) showed changes in the structures of 70–80% of the cells. The external capsule of proteinaceous material surrounding the wall of non-treated *L. paracasei* A13 cells was no longer visible after HPH treatment and the cell surface appeared jagged (indicated using an arrow in Figure 1C). Moreover, the cytoplasm appeared to be compressed and it was detached from the outermost cellular structures (indicated using an arrow in Figure 1D). The effects of pressure treatment on the outermost cellular structures were also visible in the TEM images of *L. acidophilus*, a species that is characterized by the presence of an S-layer surrounding the cell wall. As shown in Figures 2A,B, a continuous, thin, electron-dense layer was visible at the outer edge of the walls of the *L. acidophilus* DRU control cells, whereas this layer appeared discontinuous in the pressure-treated cells (Figures 2C,D; indicated using arrows). The HPH-treatment induced morphological changes observed in the TEM images did not significantly impaired cell viability, as demonstrated by cell counts obtained after HPH treatment. However, these morphological changes could be related to changes in some of the technological and functional properties of the strains observed after pressure treatment that were reported by Tabanelli et al. (2012, 2013).

### Whole cell MALDI-TOF MS fingerprinting profile

A proteomic approach using MALDI-TOF MS-based protocols was used to investigate the effect of HPH treatment and the subsequent incubation on the peptide profiles of *L. acidophilus* DRU and *L. paracasei* A13 cells.

**Figure 3:**
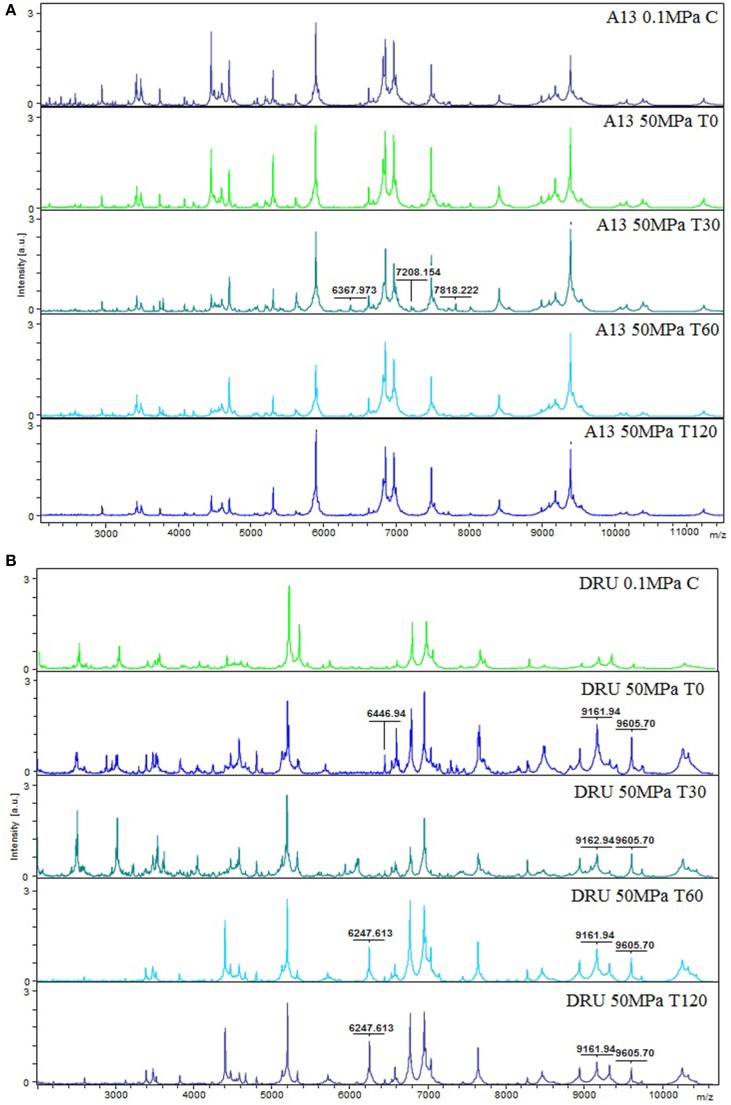
**MS proteomic profiling of ***L. paracasei*** A13 (A) and ***L. acidophilus*** DRU (B) showing MS fingerprinting of the sample conditions (0.1 MPa C, 50 MPa T0, 50 MPa T30, 50 MPa T60, and 50 MPa T120)**. The m/z-values are expressed in Da and the amplitudes are reported in a scale of intensity 10^4^ arbitrary units (a.u.). Legend: (a) A13 0.1 MPa C, (b) A13 50 MPa T0, (c) A13 50 MPa T30, (d) A13 50 MPa T60, (e) A13 50 MPa T120, (f) DRU 0.1 MPa C, (g) DRU 50 MPa T0, (h) DRU 50 MPa T30, (i) DRU 50 MPa T60, (j) DRU 50 MPa T120.

Figure 3A shows the peptide spectra of *L. paracasei* A13 cells. Differences in the peptide profiles of the 0.1 MPa C cells compared to that of the treated A13 samples were observed, particularly in the low-mass spectral region (2000–3000 Da). Increasing the incubation period to 60 min after HPH treatment increased the signals in the region between 3500 and 5200 Da. However, the 50 MPa T30 samples showed characteristic peaks at 6367.97, 7818.22, and 7818.22 Da that were absent in cells under the other conditions. Further increasing the incubation period to 120 min decreased the intensity of the peaks in the region between 3000 and 5500 Da.

Figure 3B shows the peptide spectral profiles of *L. acidophilus* DRU cells. The spectral profiles of the 0.1 MPa C cells and the 50 MPa T0 and 50 MPa T30 cells included more peptide peaks, particularly in the 2000–5000 Da region, compared with the spectral profiles of 50 MPa T60 and 50 MPa T120 samples. In contrast, the profiles of treated cells had peaks of higher intensity in the mass range of 7000–10300 Da compared with those in the profile of untreated cells. In addition, the profiles of the treated cells were characterized by the presence of peaks (i.e., at 6247.613, 6446.94, 9161.94, and 9605.68 Da) that were absent in the profiles of the control cells. Moreover, the intensity of the peaks in the 6951.51 and 7641.54 Da region of the spectra of the treated cells differed from that of the control-cell spectra.

**Figure 4:**
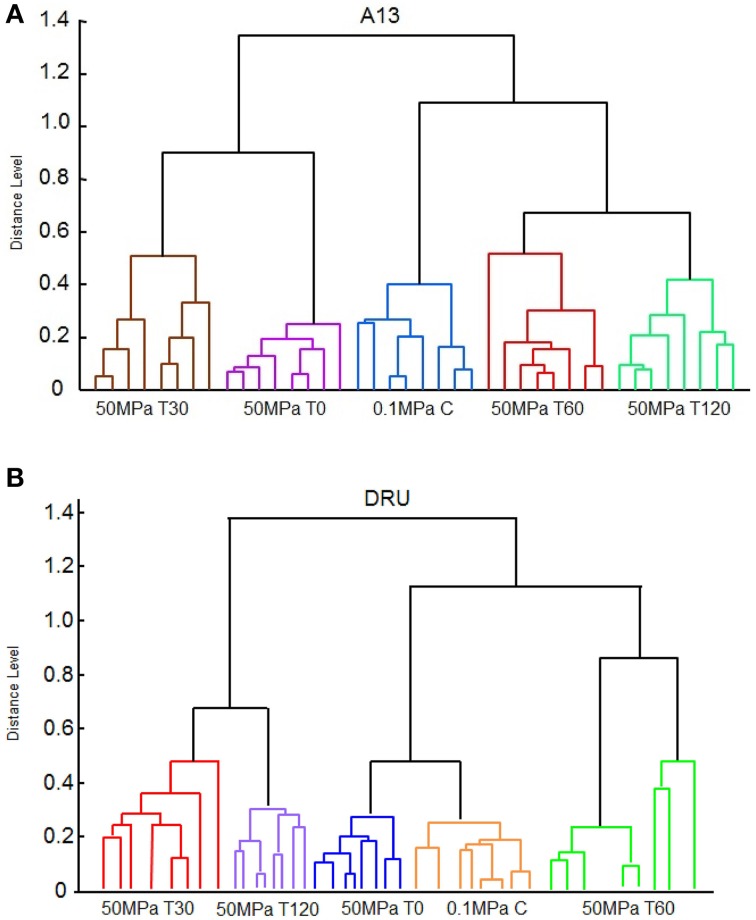
**Reference spectra dendrogram obtained by principal component dendrogram (PCA) analyses for ***Lactobacillus paracasei*** A13 (A) and for ***Lactobacillus acidophilus*** DRU (B)**.

The dendrograms that were derived using the MALDI-TOF MS Biotyper profiles showed clusters associated with HPH treatment and with the period of incubation at 37°C. In particular, the dendrogram of the spectra of *L. paracasei* A13 cells showed two major clusters (Figure 4A). The first cluster grouped the *L. paracasei* A13 50 MPa T0 and 50 MPa T30 cells. The second major cluster grouped 0.1 MPa C cells and 50 MPa T60, and 50 MPa T120 cells, meaning that the peptide profiles of the untreated cells and the treated cells that were incubated for 60 or 120 min were less than 0.8 apart. However, all of the tested conditions were well differentiated.

The spectral dendrograms of *L. acidophilus* DRU cells showed two major clusters, as follows: the first cluster included 50 MPa T30 and 50 MPa T120 cells and the second cluster included the 0.1 MPa C, 50 MPa T0, and 50 MPa T60 cells (Figure 4B). The last cluster could be sub-grouped into two minor clusters in which the control cells and the cells analyzed immediately after the hyperbaric treatment were grouped together, whereas 50 MPa T60 cells were in a separate cluster. In the case of this strain, all of the tested conditions were also well distinguished by their associated spectra.

Pearson's correlation analysis showed a high level of reproducibility of the data for *L. paracasei* A13 cells, ranging from 0.89 to 0.98, whereas the level of reproducibility of the data for *L. acidophilus* cells was slightly lower, ranging from 0.71 to 0.97.

### Evaluation of the effects of HPH treatment and the medium pH value on the volatile profiles of buttermilk

To study the effects of HPH treatment on the volatile profiles of probiotic-containing buttermilk samples (at pH 7 or pH 4.6), buttermilk was inoculated with *L. acidophilus* DRU or *L. paracasei* A13 cells at a concentration of approximately 8 log CFU/ml and was passed through a high pressure homogenizer at 0.1 MPa (control) or at 50 MPa (HPH treated). All of the samples were stored at 4°C for 30 days. The aroma profiles and cell viability rates were monitored throughout the storage period. The viability results confirmed that buttermilk supported the survival of adequate numbers of probiotic LAB cells during refrigerated storage, independently of the level of HPH treatment, as demonstrated previously (Burns et al., 2008b; Tabanelli et al., 2013). The viability rates of the two strains remained greater than 7.6 log CFU/ml at both pH levels throughout the refrigerated storage period.

The content of the compounds detected in the aroma profile of uninoculated buttermilk and buttermilk samples that were inoculated with *L. paracasei* A13 and *L. acidophilus* DRU cells were expressed as the % of the total peak area, as shown in Tables 1–3, respectively.

**Table 1:** **Volatile compounds (expressed as % peak area) detected in uninoculated buttermilk after 15 and 30 days of refrigerate storage**.

**Compounds**	**Uninoculated buttermilk**		
	**0 days**	**15 days**	**30 days**
Hexanal	0.61 (±0.07)	4.73 (±0.23)	3.39 (±0.32)
Furfural	2.68 (±0.17)	2.92 (±0.13)	2.67 (±0.21)
Benzaldehyde	2.35 (±0.19)	1.86 (±0.17)	2.05 (±0.13)
Octanal	–^*^	0.60 (±0.07)	–
**Aldehydes**	**5.65**	**10.12**	**8.11**
2-propanone	7.40 (±0.39)	8.62 (±0.47)	6.59 (±0.42)
2-butanone	2.01 (±0.11)	2.26 (±0.15)	2.27 (±0.12)
1- hydroxy -2-propanone	1.23 (±0.03)	2.79 (±0.13)	2.37 (±0.21)
2-pentanone	15.69 (±0.78)	13.64 (±0.67)	15.06 (±0.96)
2-heptanone	42.78 (±1.39)	35.55 (±1.56)	37.71 (±1.23)
3,5-octadien-2-one	0.66 (±0.07)	0.30 (±0.02)	0.29 (±0.06)
2-nonanone	6.49 (±0.29)	5.08 (±0.11)	5.68 (±0.38)
2-undecanone	0.86 (±0.02)	0.85 (±0.05)	0.87 (±0.06)
1-phenyl-ethanone	1.88 (±0.05)	1.34 (±0.08)	1.53 (±0.09)
**Ketons**	**79.01**	**70.43**	**72.38**
2-furanmethanol	11.42 (±0.69)	13.87 (±0.17)	14.84 (±0.88)
**Alcohols**	**11.42**	**13.87**	**14.84**
**Acetic acid**	3.34 (±0.28)	5.11 (±0.36)	4.14 (±0.21)
Hexanoic acid	0.59 (±0.02)	0.48 (±0.03)	0.53 (±0.06)
Acids	**3.93**	**5.59**	**4.68**

* *Under detection limit. The data are mean of six repetitions. The standard deviation is reported within brackets*.

**Table 2:** **Volatile compounds (expressed as % peak area) detected in buttermilk at pH 4.6 and 7, inoculated with *Lactobacillus paracasei* A13 treated at 0.1 and 50 MPa, after 15 and 30 days of refrigerate storage**.

**Compounds**	**15 days**				**30 days**			
	**pH 4.6**		**pH 7.0**		**pH 4.6**		**pH 7.0**	
	**0.1 MPa**	**50 MPa**	**0.1 MPa**	**50 MPa**	**0.1 MPa**	**50 MPa**	**0.1 MPa**	**50 MPa**
Hexanal	1.61 (±0.12)	0.58 (±0.03)	1.51 (±0.09)	–^*^	–	–	1.99 (±0.12)	–
Furfural	2.29 (±0.13)	2.06 (±0.17)	3.30 (±0.14)	4.22 (±0.35)	4.72 (±0.28)	1.25 (±0.78)	2.32 (±0.13)	2.56 (±0.11)
Benzaldehyde	0.89 (±0.03)	1.16 (±0.09)	1.87 (±0.11)	1.96 (±0.12)	1.17 (±0.08)	0.77 (±0.02)	2.52 (±0.18)	1.20 (±0.09)
4-methyl-benzaldehyde	3.25 (±0.28)	3.07 (±0.19)	4.98 (±0.23)	6.65 (±0.47)	3.38 (±0.29)	2.33 (±0.11)	5.91 (±0.27)	4.73 (±0.14)
Non-anal	4.67 (±0.32)	–	–	–	–	2.77 (±0.20)	–	–
**Aldehydes**	**12.71**	**6.87**	**11.66**	**12.82**	**9.27**	**7.11**	**12.74**	**8.50**
2-propanone	8.44 (±0.38)	5.19 (±0.23)	13.13 (±0.51)	8.81 (±0.41)	4.36 (±0.38)	5.06 (±0.31)	19.69 (±1.07)	6.25 (±0.21)
2-butanone	4.30 (±0.18)	7.63 (±0.47)	5.32 (±0.27)	9.16 (±0.44)	2.14 (±0.11)	5.85 (±0.29)	5.58 (±0.32)	7.97 (±0.35)
2,3-butanedione	–	–	–	2.24 (±0.15)	–	–	–	5.63 (±0.22)
3-hydroxy-2-butanone	–	–	–	–	–	–	–	6.11 (±0.46)
1-hydroxy-2-propanone	1.38 (±0.05)	0.62 (±0.05)	0.97 (±0.03)	1.06 (±0.06)	1.80 (±0.07)	–	0.89 (±0.02)	0.88 (±0.09)
2-pentanone	3.04 (±0.29)	3.36 (±0.13)	6.95 (±0.26)	4.05 (±0.21)	3.59 (±0.28)	1.62 (±0.11)	6.93 (±0.28)	3.11 (±0.18)
2-heptanone	4.11 (±0.28)	7.95 (±0.61)	16.32 (±1.56)	8.32 (±0.78)	9.18 (±0.39)	5.03 (±0.58)	10.67 (±0.43)	11.99 (±0.57)
3,5-octadien-2-one	–	0.37 (±0.03)	0.57 (±0.04)	0.84 (±0.05)	0.96 (±0.07)	0.41 (±0.04)	5.41 (±0.35)	6.06 (±0.37)
2 non-anone	1.55 (±0.08)	2.89 (±0.18)	6.66 (±0.27)	8.24 (±0.59)	3.15 (±0.20)	1.88 (±0.11)	–	–
2-undecanone	0.17 (±0.03)	0.69 (±0.04)	1.38 (±0.07)	1.87 (±0.13)	0.45 (±0.03)	0.49 (±0.01)	0.81 (±0.02)	1.41 (±0.11)
**Ketons**	**22.99**	**28.70**	**51.29**	**44.59**	**25.64**	**20.34**	**49.98**	**49.41**
Ethyl alcohol	1.49 (±0.10)	2.03 (±0.13)	2.67 (±0.16)	3.51 (±0.28)	–	1.77 (±0.11)	2.84 (±0.21)	1.61 (±0.12)
1-pentanol	–	1.22 (±0.08)	–	1.24 (±0.03)	–	1.14 (±0.07)	1.73 (±0.13)	1.17 (±0.09)
1-hexanol	–	–	–	1.20 (±0.09)	–	0.38 (±0.03)	0.96 (±0.08)	0.72 (±0.07)
2-furanmethanol	13.24 (±0.96)	11.25 (±0.83)	17.79 (±0.91)	19.17 (±1.56)	18.97 (±1.02)	11.83 (±0.96)	17.48 (±1.36)	17.78 (±1.22)
1-nonenol	–	–	3.52 (±0.29)	3.45 (±0.28)	–	–	–	0.79 (±0.08)
**Alcohols**	**14.73**	**14.50**	**23.98**	**28.57**	**18.97**	**15.12**	**23.02**	**22.08**
Acetic acid	26.20 (±1.30)	24.42 (±1.16)	7.87 (±0.25)	9.20 (±0.37)	27.42 (±1.33)	30.06 (±1.68)	9.83 (±0.53)	14.44 (±1.01)
Hexanoic acid	13.21 (±0.84)	13.12 (±0.91)	2.55 (±0.13)	2.10 (±0.16)	11.39 (±0.59)	14.12 (±0.77)	3.01 (±0.23)	3.39 (±0.26)
Butanoic acid	10.16 (±0.86)	9.84 (±0.65)	2.65 (±0.21)	1.50 (±0.11)	7.32 (±0.47)	11.06 (±0.86)	1.43 (±0.07)	2.18 (±0.18)
**Acids**	**49.58**	**47.37**	**13.07**	**12.80**	**46.12**	**55.24**	**14.27**	**20.01**
Ethylacetate	–	2.56 (±0.19)	–	4.71 (±0.21)	–	2.19 (±0.18)	–	–
**Esters**	**0.00**	**2.56**	**0.00**	**4.71**	**0.00**	**2.19**	**0.00**	**0.00**

* *Under detection limit. The data are mean of six repetitions. The standard deviation is reported within brackets*.

**Table 3:** **Volatile compounds (expressed as % peak area) detected in buttermilk at pH 4.6 and 7, inoculated with *Lactobacillus acidophilus* DRU treated at 0.1 and 50 MPa, after 15 and 30 days of refrigerate storage**.

**Compounds**	**15 days**				**30 days**			
	**pH 4.6**		**pH 7.0**		**pH 4.6**		**pH 7.0**	
	**0.1 MPa**	**50 MPa**	**0.1 MPa**	**50 MPa**	**0.1 MPa**	**50 MPa**	**0.1 MPa**	**50 MPa**
Hexanal	2.91 (±0.28)	2.95 (±0.15)	3.16 (±0.18)	4.63 (±0.32)	1.29 (±0.10)	–^*^	3.28 (±0.15)	–
Furfural	2.72 (±0.19)	2.78 (±0.11)	3.47 (±0.22)	2.86 (±0.14)	2.79 (±0.13)	0.49 (±0.04)	3.81 (±0.29)	0.76 (±0.04)
Benzaldehyde	1.74 (±0.16)	2.15 (±0.16)	2.87 (±0.26)	2.41 (±0.16)	2.42 (±0.15)	0.08 (±0.01)	3.90 (±0.35)	–
Octanal	0.46 (±0.02)	0.91 (±0.14)	0.51 (±0.04)	0.48 (±0.03)	–	–	–	–
4-methyl, benzaldehyde	4.37 (±0.35)	2.33 (±0.20)	5.35 (±0.51)	2.76 (±0.13)	4.43 (±0.30)	0.42 (±0.03)	5.70 (±0.28)	0.84 (±0.06)
Non-anal	1.40 (±0.10)	1.16 (±0.08)	1.22 (±0.06)	1.30 (±0.09)	–	–	–	–
**Aldehydes**	**13.60**	**12.29**	**16.58**	**14.44**	**10.93**	**0.98**	**16.70**	**1.61**
2-propanone	7.19 (±0.42)	5.09 (±0.39)	6.34 (±0.40)	5.80 (±0.53)	9.24 (±0.44)	2.49 (±0.11)	11.72 (±0.88)	2.46 (±0.13)
2-butanone	3.39 (±0.19)	8.32 (±0.61)	4.39 (±0.16)	10.76 (±0.80)	4.42 (±0.31)	3.38 (±0.36)	7.19 (±0.35)	4.45 (±0.39)
2-pentanone	3.45 (±0.23)	3.15 (±0.20)	4.85 (±0.38)	5.95 (±0.42)	3.93 (±0.25)	23.34 (±1.86)	6.44 (±0.28)	6.60 (±0.47)
2-heptanone	9.72 (±0.45)	8.77 (±0.68)	23.03 (±1.16)	14.87 (±0.56)	9.01 (±0.74)	39.15 (±2.04)	8.31 (±0.51)	30.25 (±1.74)
3,5-octadien-2-one	0.47 (±0.02)	0.34 (±0.02)	0.56 (±0.04)	0.20 (±0.01)	0.29 (±0.02)	–	0.59 (±0.03)	–
2-nonanone	3.56 (±0.27)	3.12 (±0.15)	4.88 (±0.40)	4.79 (±0.27)	3.78 (±0.28)	10.85 (±0.84)	6.54 (±0.44)	20.53 (±1.33)
2-undecanone	0.90 (±0.07)	0.68 (±0.04)	0.96 (±0.06)	1.67 (±0.14)	0.52 (±0.03)	0.68 (±0.07)	1.23 (±0.16)	0.76 (±0.10)
**Ketons**	**28.69**	**29.47**	**45.00**	**44.03**	**31.19**	**79.88**	**42.01**	**65.05**
Ethyl alcohol	–	1.49 (±0.13)	0.68 (±0.04)	1.59 (±0.11)	1.67 (±0.09)	0.99 (±0.06)	1.64 (±0.15)	1.58 (±0.11)
2-heptanol	–	–	0.86 (±0.06)	0.76 (±0.05)	–	0.98 (±0.04)	–	1.58 (±0.13)
2-furanmethanol	15.19 (±1.13)	12.21 (±0.71)	16.32 (±0.86)	13.98 (±1.06)	13.22 (±0.83)	4.30 (±0.18)	16.26 (±0.95)	3.59 (±0.20)
**Alcohols**	**15.19**	**13.69**	**17.85**	**16.33**	**14.89**	**6.27**	**17.89**	**6.75**
Acetic acid	6.81 (±0.53)	7.23 (±0.32)	0.94 (±0.06)	0.77 (±0.06)	5.23 (±0.44)	1.58 (±0.11)	1.04 (±0.07)	0.28 (±0.03)
Butanoic acid	10.99 (±0.56)	11.35 (±1.06)	–	–	6.26 (±0.35)	2.75 (±0.18)	–	–
Hexanoic acid	9.38 (±0.55)	8.64 (±0.55)	0.63 (±0.04)	0.56 (±0.02)	8.73 (±0.51)	1.84 (±0.12)	3.03 (±0.15)	0.63 (±0.04)
**Acids**	**27.18**	**27.22**	**1.57**	**1.32**	**20.22**	**6.17**	**4.07**	**0.91**
Ethylacetate	0.15 (±0.01)	3.62 (±0.15)	1.14 (±0.06)	7.56 (±0.70)	7.88 (±0.61)	0.42 (±0.03)	1.43 (±0.13)	10.31 (±0.76)
Ethylhexanoate	–	–	–	–	–	–	–	8.62 (±0.53)
**Esters**	**0.15**	**3.62**	**1.14**	**7.56**	**7.88**	**0.42**	**1.43**	**18.93**

* *Under detection limit. The data are mean of six repetitions. The standard deviation is reported within brackets*.

The most abundant molecules detected in the non-inoculated and untreated buttermilk samples were ketones, such as 2-propanone, 2-butanone, 2-pentanone, and 2-heptanone, aldehydes, such as hexanal, furfural, and benzaldehyde, alcohols, such as hexanol and 2-furanmethanol, and acetic acid (Table 1).

As expected, the aroma profiles of the buttermilk samples containing cells of the two strains studied were quite different from the profiles of the control samples due to microbial activity. However, In the case of the samples containing *L. paracasei* A13 cells, the differences between the samples at 15 and 30 days of storage were not pronounced. All the samples at pH 7 were characterized by a higher content of ketones, mainly 2-nonanone, 2-pentanone, and 2-heptanone. Significant levels of 2,3-butanedione and 3 hydroxy-2-butanone were also detected in the HPH-treated samples at pH 7 at 30 days. Alcohols, mainly furanmethanol, were more abundant in buttermilk samples at pH 7, whereas acids (mainly acetic acid) predominated at pH 4.6, reaching relative proportions that were always greater than 45%. The content of esters in the samples containing *L. paracasei* A13 cells was always negligible, unlike that of samples containing *L. acidophilus* DRU cells.

Aldehydes accounted for approximately 15% of the total peak area of the volatile molecule profile of the *L. acidophilus* DRU-containing samples after 15 days of refrigerated storage, with no significant differences due to the pH value or HPH treatment, and the most prominent of these compounds were hexanal, 4-methyl-benzaldehyde, benzaldehyde and furfural. However, the levels of these compounds in the non-treated samples remained stable during 30 days of storage, whereas their levels in the HPH-treated buttermilk samples fell down below 2% during this period. Ketones were the most prominent chemical group present under all of the conditions, but significant differences were observed in relation to HPH treatment and the pH level. At 15 days of storage, the total ketones represented approximately 29 and 45% of the total peak area at pH 4.6 and 7, respectively, with no differences related to HPH treatment. The higher level of total ketones in buttermilk at pH 7 was mainly due to the higher concentration of 2-heptanone compared to the buttermilk at pH 4.6. The content of 2-butanone was higher in the treated samples, whereas the level of 2-propanone was higher in the control samples. 2-pentanone and 2-nonanone were detected in significant and constant concentrations in all of the samples. At 30 days of storage, the ketone concentrations in the non-treated control buttermilk were little changed, but those of the HPH-treated buttermilks had drastically increased, largely due to increases in the relative concentrations of 2-pentanone, 2-heptanone, and 2-nonanone. The alcohols present were mainly represented by furanmethanol, the concentration of which was higher in the non-treated buttermilk sample than in the HPH-treated buttermilk samples at 15 days of storage. This difference was significantly greater at 30 days of storage regardless of the pH value. In contrast, the pH value strongly affected the relative percentage of acids, the levels of which were higher at pH 4.6 than at pH 7, independently of HPH treatment and the storage period. Finally, a significantly higher concentration of ethyl acetate was observed in HPH-treated buttermilk at pH 7 at 15 days of storage. The level of this ester was increased at 30 days of storage and was accompanied by an increased level of ethyl hexanoate.

### Principal component analysis on the volatile profiles of buttermilk

**Figure 5:**
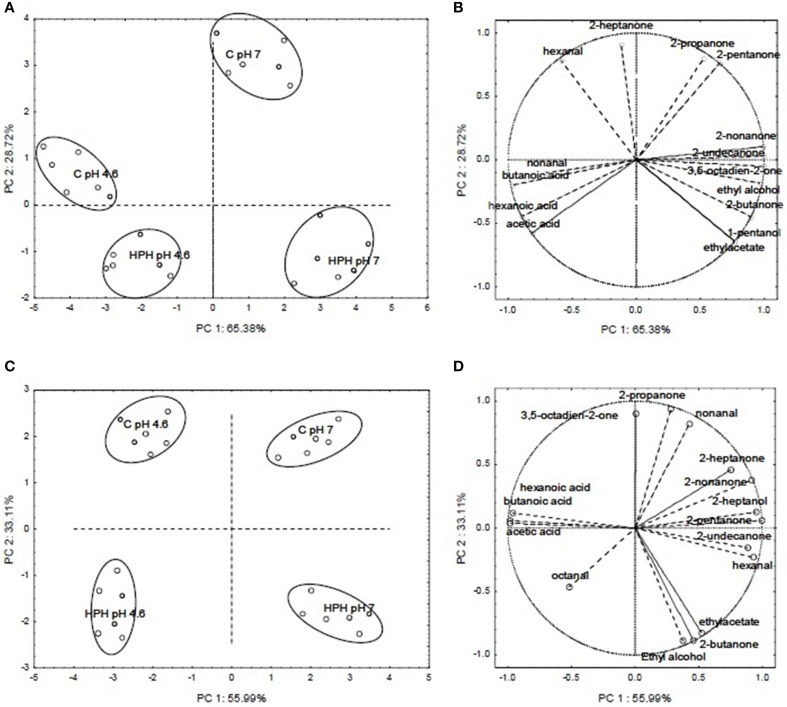
**PCA loading plots and variable factor coordinates for the two principal components relative to the aroma compounds of control or HPH-treated buttermilk inoculated with Lactobacillus paracasei A13 (A,B) and Lactobacillus acidophilus DRU (C,D) after 15 days of storage at 4°C**. In PCA loading plots (A,C) the different samples are indicated by letters, an namely: C pH 7 (not acidified control samples), C pH 4.6 (acidified control samples), HPH pH 7 (not acidified treated samples) and HPH pH 4.6 (acidified treated samples).

To better evaluate the effect of HPH treatment on the aroma profile of buttermilk, a Principal Component Analysis (PCA) was conducted using the % of the peak area of the volatile compounds listed in Tables 1, 2. Figures 5A,C show PCA loading plots of the aroma profile data for buttermilk that was inoculated with *L. paracasei* A13 cells or *L. acidophilus* DRU cells, respectively, which demonstrated that the first two principal components (PC1 and PC2) explained more than 85% of the total variability. In both cases, PC1 accounted for the greater part of the variability (approximately 65.38 and 55.99% for the *L. paracasei* A13-containing samples and the *L. acidophilus* DRU-containing samples, respectively), and the samples could be grouped into four clusters according to whether they were HPH treated and the pH of the medium. The control buttermilk samples were grouped in the upper part of the plot, whereas the HPH-treated samples were grouped in the lower part. Moreover, the acidified samples were grouped on the left side of the plot, and the pH 7 buttermilk samples were grouped on the right side.

Figure 5B shows the variable-factor coordinates for the first two principal components for the L. paracasei A13 samples. The acidified (pH 4.6) control samples were characterized by the presence of non-anal and acids, whereas the pH 7 control samples were characterized mainly by the presence of ketones, such as 2-heptanone, 2-propanone, and 2-pentanone. The variable factor coordinates for the first two principal components of the buttermilk samples that were inoculated with L. acidophilus DRU cells are shown in Figure 5D. As was the case for the control samples, the acidified L. acidophilus DRU-containing samples were characterized mainly by the presence of acids, whereas the untreated samples and the HPH-treated samples at pH 7 were characterized mainly by the presence of 2-heptanone and 2-nonanone and the presence of ethyl acetate, 2-butanone and ethyl alcohol, respectively.

## Discussion

In previous studies, it was demonstrated that HPH treatment at 50 MPa enhanced certain probiotic properties of LAB and changed the fatty acid composition of the cell membrane as response to the sub-lethal stress applied (Tabanelli et al., 2013, 2014). Moreover, the HPH treatment significantly reduced the hydrophobicity of the L. acidophilus DRU cells, whereas this treatment increased that of the L. paracasei A13 cells by five-fold. Tabanelli et al. (2012) correlated these differences with the different gastrointestinal-transit behaviors and gut-epithelial interactions shown by these two probiotic strains in mice. Furthermore, the hydrophobicity of the cells was found to correlate with their adhesive ability (Basson et al., 2007) and changes in the outermost cellular structures, following HPH treatment, could influence the intestinal transit and behavior of the tested strains. TEM micrographs demonstrated the effect of HPH treatment on the cell-wall structures, which was strain-dependent. The TEM images of the control samples (0.1 MPa-treated) of L. acidophilus, a species characterized by the presence of an S-layer, showed an intact layer surrounding each cell. Tabanelli et al. (2013) demonstrated that the cells of this strain had a higher level of in vitro hydrophobicity compared to that of L. paracasei A13 cells and attributed this property to this additional external proteinaceous structure. Furthermore, several of the cell-surface proteins of the S-layer, which represent approximately 10% of the total cellular proteins, were reported to have adhesion domains and to be involved in cell adhesion (Åvall-Jääskeläinen and Palva, 2005; Jakava-Viljanen and Palva, 2007). After HPH treatment, the layer surrounding the wall of L. acidophilus DRU cells was discontinuous, which could account for the loss of hydrophobicity observed in this strain after pressure treatment by Tabanelli et al. (2013). Additionally, it is evident that factors and treatments that modify the outermost cellular structures, such as HPH, could affect the functional properties of probiotic strains. In several lactobacilli species, such as L. crispatus and L. acidophilus, the removal or damage of the S-layer proteins resulted in a decreased ability to bind to the epithelium of the host (Buck et al., 2005; Frece et al., 2005).

TEM micrographs of L. paracasei A13 cells subjected to HPH treatment showed many changes in the outermost cellular structures (i.e., proteinaceous material that normally surrounding the cell wall was no longer visible). These changes could be responsible for the increased hydrophobicity of the cells observed by Tabanelli et al. (2013) following HPH treatment.

Although analysis of MALDI-TOF spectra did not permit the identification of the MS/MS peptides, specific peptide fingerprints associated with the strain, the HPH treatment and the incubation period were obtained using this technique. The proteomic profiles of cells of the probiotic strains L. paracasei A13 and L. acidophilus DRU that were treated using HPH and were incubated at 37°C for different periods showed peptide patterns different from those of untreated cells. These differences can most likely be attributed to the effect of HPH on the cell-surface proteins and the cellular response to the HPH treatment. However, L. paracasei A13 and L. acidophilus DRU cells showed different behaviors independently of the HPH treatment and the incubation period that appeared to be related to their differential responses to the applied stress.

In particular, the dendrograms associated with *L. paracasei* A13 cells demonstrated that the peptide profiles of treated cells obtained immediately after the hyperbaric treatment were significantly different from those of the control cells, whereas the differences diminished during the incubation period. In fact, the peptide profiles of treated cells incubated for 60 and 120 min were more similar to that of the control cells. The changes in the peptide profiles of treated cells after 30 min of incubation with respect to the peptide profiles of the control cells can be attributed to the presence of specific enzymes and stress proteins that are involved in the restoration of the status *quo ante*.

In contrast, the analysis of the dendrograms associated with *L. acidophilus* DRU cells showed that the peptide profiles of these cells obtained immediately after pressure treatment were more similar to those of the control cells than were the profiles obtained later, showing that the HPH treatment had less effect on this strain than on *L. paracasei* A13, most likely due to the higher resistance to physical stresses conferred by the S-layer. However, the hyperbaric treatment caused significant modifications of the peptide profile that became evident during the incubation period, indicating that the HPH treatment induced persistent metabolic changes. The lack of peptide identification did not allow distinguishing among the released and novel peptides. However, in the case of *L. paracasei* A13, the similarity of the peptide spectrum of the control cells and that of the treated cells immediately after hyperbaric treatment, as well as the appearance of characteristic peaks during the incubation of the treated cells, suggested that both released and novel peptides contributed to the profile changes that were observed over time. In contrast, in the case of *L. acidophilus* DRU, the similarity of the spectra of the control cells and those of the cells immediately following the HPH treatment suggested that the synthesis of novel proteins was a key mechanism in the stress response of this S-layer-endowed strain.

It is well-known that exposure to physico-chemical stresses results in increased levels of synthesis of stress-response proteins. Jofré et al. (2007) showed that after 2 h of recovery from high hydrostatic-pressure treatment (HHP), several Gram-positive strains expressed transcription factors and proteins related to the synthesis of enzymes involved in energy metabolism. In addition, pressure application (both HHP and HPH) was reported to cause conformational changes in proteins, protein unfolding and the dissociation of oligomeric or aggregated proteins while also affecting enzymatic activities (Fantin et al., 1996; Vannini et al., 2008). In particular, HPH treatment was reported to cause protein conformational changes as well as protein aggregation and to affect the interactions of proteins with other macromolecules, such as polysaccharides and lipids (Floury et al., 2000; Patrignani et al., 2009). The effects of HPH on microorganisms could be attributed to the following: (I) a direct effect of the pressure exerted on the integrity of the cell wall or the outer membranes; (II) the passage of proteins through the damaged cell walls and membranes; and (III) indirect stimulatory effects on the functions of proteins caused by small structural changes that affect their active sites (Diels and Michiels, 2006).

The analysis of the aroma profiles of the two different species showed that they had specific fingerprints (i.e., acetic acid was more pronounced in the profile of the *L. paracasei* samples). Moreover, the pH value of the medium affected the volatile profiles through affecting the metabolic pathways of the bacteria and the level of activity of their enzymes. In addition, the pH value was shown to alter the volatility of compounds such as acids through affecting their interactions with the buttermilk matrix as well as the water-binding capacity of the proteins present (Innocente et al., 2011). In particular, 2-propanone, 2-butanone, furfural, and furanmethanol were detected at both 15 and 30 days of refrigerated storage of buttermilk prepared using any of the samples of both strains under any of the conditions tested. In buttermilk with a pH value of 7 that was inoculated with *L. paracasei* A13 cells and HPH treated at 50 MPa, acetoin was detected at 30 days of storage and diacetyl (2, 3-butanone) was detected at 15 and 30 days of storage. Lanciotti et al. (2007a) showed an increase in the content of several molecules in the aroma profiles of dairy products containing the cells of several *Lactobacillus* species that had been directly treated using a sub-lethal HPH level. Additionally, Patrignani et al. (2007) reported that increasing the level of HPH treatment of probiotic-containing fermented milks increased the diacetyl content. Several alcohols (ethanol, non-anol, 3-methyl-1-butanol, and 2-ethyl-hexanol), acids (butanoic, heptanoic, and decanoic acids) and ethyl-esters (ethylacetate, ethylbutanoate, ethylhexanoate, ethylheptanoate, and ethyldecanoate) were detected in inoculated samples compared to untreated and un-inoculated buttermilk, although in different amounts in relation to the strain used and treatment applied.

The significant differences in the volatile-molecule profiles of these samples could be due to the effects of the HPH stress on the microbial cells. In particular, the increase in the content of ketones and esters observed after pressure treatment could be associated with higher levels of activity of lipases and esterases. There is much evidence showing that sub-lethal HPH treatment affects the membrane fatty-acid desaturase enzymes that are involved in the active response of cells to high-pressure stress (Somero, 1992; Guerzoni et al., 1997; Tabanelli et al., 2014). In addition, HPH treatment has been reported to alter the activity of several enzymes of microbial origin as well as some of those that naturally occur in food matrices (Vannini et al., 2004; Iucci et al., 2006; Lanciotti et al., 2007b). Moreover, Patrignani et al. (2013) showed increased levels of esters and ketones in yeast cells subjected to HPH treatment in fruit juice. The involvement of ketones in the stress-response mechanisms of microbial cells was documented, whereas esters were regarded as yeast signaling molecules (Isakoff et al., 1996; Kocsis and Weselake, 1996).

The results of the present study demonstrated overall changes in the aroma profile and the production of molecules that positively affected the sensory profile of probiotic cell-containing buttermilk samples that were pressure treated. Therefore, because probiotic products manufactured using only probiotic strains are often characterized by the lack of desirable sensory features or a homogeneous aroma profile, treating probiotic cells with HPH might differentiate the products and enhance their positive sensory properties.

In addition, the results obtained suggested that HPH has several biotechnological applications, including modulating the volatile-molecule profiles of dairy products, improving specific enzymatic activities of cells and enhancing the probiotic properties of bacterial strains. Finally, the proteomic approach used in this study has contributed to add another dowel to the understanding of the mechanisms underlying the stress responses of probiotic strains by demonstrating the involvement of the peptide profile in the response to HPH, which is one of the most promising technologies for application at the industrial level, particularly in the dairy-product sector.

However, the promising aspects of HPH treatment indicated by the results obtained in this study must be further investigated to better understand the relationships among the genomic, volatilomic and peptide-metabolic profiles.

### Conflict of interest statement

The authors declare that the research was conducted in the absence of any commercial or financial relationships that could be construed as a potential conflict of interest.

## References

[B1] Åvall-Jääskeläinen S. Palva A. (2005). Lactobacillus surface layers and their applications. FEMS microbial. Rev. 29, 511–529. 10.1016/j.femsre.2005.04.003 15935509

[B2] Basson A. Flemming L. A. Chenia H. Y. (2007). Evaluation of adherence, hydrophobicity, aggregation, and biofilm development of Flavobacterium johnsoniae-like isolates. Microb. Ecol. 55, 1–14. 10.1007/s00248-007-9245-y 17401596

[B3] Buck B. L. Altermann E. Svingerud T. Klaenhammer T. R. (2005). Functional analysis of putative adhesion factors in Lactobacillus acidophilus NCFM. Appl. Environ. Microbiol. 71, 8344–8355. 10.1128/AEM.71.12.8344-8351.2005 16332821 PMC1317474

[B4] Burns P. Patrignani F. Serrazanetti D. Vinderola G. Reinheimer J. Lanciotti R.. (2008a). Probiotic Crescenza cheese containing Lactobacillus paracasei and Lactobacillus acidophilus manufactured with high pressure-homogeneized milk. J. Dairy Sci. 91, 500–512. 10.3168/jds.2007-0516 18218736

[B5] Burns P. Vinderola G. Molinari F. Reinheimer J. (2008b). Suitability of whey and buttermilk for the growth and frozen storage of probiotic lactobacilli. Int. J. Dairy Technol. 61, 156–164. 10.1111/j.1471-0307.2008.00393.x

[B6] Bury D. Jelen P. Kaláb M. (2001). Disruption of Lactobacillus delbrueckii ssp. bulgaricus 11842 cells for lactose hydrolysis in dairy products: a comparison of sonication, high-pressure homogenization and bead milling. Innov. Food Sci. Emerg. 2, 23–29. 10.1016/S1466-8564(00)00039-4

[B7] Croxatto A. Prod'Hom G. Greub G. (2012). Applications of MALDI-TOF mass spectrometry in clinical diagnostic microbiology. FEMS Microbial. Rev. 36, 380–407. 10.1111/j.1574-6976.2011.00298.x 22092265

[B8] Diels A. M. Michiels C. W. (2006). High-pressure homogenization as a non-thermal technique for the inactivation of microorganisms. Crit. Rev. Microbiol. 32, 201–216. 10.1080/10408410601023516 17123905

[B9] Fantin G. Fogagnolo M. Guerzoni M. E. Lanciotti R. Medici A. Pedrini P. (1996). Effect of high hydrostatic pressure and high pressure homogenization on the enantioselectivity of microbial reduction. Tetrahedron-Asymmet. 7, 2879–2887. 10.1016/0957-4166(96)00379-5

[B10] Floury J. Desrumaux A. Lardières J. (2000). Effect of high-pressure homogenization on droplet size distributions and rheological properties of model oil-in-water emulsions. Innov. Food Sci. Emerg. 1, 127–134.

[B11] Frece J. Kos B. Svetec I. K. Zgaga Z. Mrša V. Šušković J. (2005). Importance of S-layer proteins in probiotic activity of Lactobacillus acidophilus M92. J. Appl. Microbiol. 98, 285–292. 10.1111/j.1365-2672.2004.02473.x 15659182

[B12] Guerzoni M. E. Ferruzzi M. Sinigaglia M. Criscuoli G. C. (1997). Increased cellular fatty acid desaturation as a possible key factor in thermotolerance in Saccharomyces cerevisiae. Can. J. Microbiol. 43, 569–576. 9226876 10.1139/m97-080

[B13] Innocente N. Marchesini G. Biasutti M. (2011). Feasibility of the SPME method for the determination of the aroma retention capacity of proteose-peptone milk protein fraction at different pH values. Food Chem. 124, 1249–1257. 10.1016/j.foodchem.2010.07.056

[B14] Isakoff S. J. Wang Y. Skolnik E. Y. (1996). Finally, some signaling molecules find a home in yeast. Nat. Biotechnol. 14, 578–578. 10.1038/nbt0596-578 9630942

[B15] Iucci L. Patrignani F. Vallicelli M. Guerzoni M. E. Lanciotti R. (2006). Effects of high pressure homogenization on the activity of lysozyme and lactoferrin against Listeria monocytogenes. Food Control 18, 558–565. 10.1016/j.foodcont.2006.01.005

[B16] Jakava-Viljanen M. Palva A. (2007). Isolation of surface (S) layer protein carrying Lactobacillus species from porcine intestine and faeces and characterization of their adhesion properties to different host tissues. Vet. Microbial. 124, 264–273. 10.1016/j.vetmic.2007.04.029 17544232

[B17] Jofré A. Champomier-Vergès M. Anglade P. Baraige F. Martín B. Garriga M.. (2007). Protein synthesis in lactic acid and pathogenic bacteria during recovery from a high pressure treatment. Res. Microbiol. 158, 512–520. 10.1016/j.resmic.2007.05.005 17631981

[B18] Kankaanpää P. Salminen S. J. Isolauri E. Lee Y. K. (2001). The influence of polyunsaturated fatty acids on probiotic growth and adhesion. FEMS Microbiol. Lett. 194, 149–153. 10.1016/S0378-1097(00)00519-X 11164299

[B19] Kankaanpää P. Yang B. Kallio H. Isolauri E. Salminen S. (2004). Effects of polyunsaturated fatty acids in growth medium on lipid composition and on physicochemical surface properties of lactobacilli. Appl. Environ. Microbiol. 70, 129–136. 10.1128/AEM.70.1.129-136.2004 14711634 PMC321255

[B20] Kirjavainen P. V. Ouwehand A. C. Isolauri E. Salminen S. J. (1998). The ability of probiotic bacteria to bind to human intestinal mucus. FEMS Microbiol. Lett. 167, 185–189. 10.1016/S0378-1097(98)00387-5 9809419

[B21] Kocsis M. G. Weselake R. J. (1996). Phosphatidate phosphatases of mammals, yeast, and higher plants. Lipids 31, 785–802. 10.1007/BF02522974 8869881

[B22] Lanciotti R. Patrignani F. Iucci L. Guerzoni M. E. Suzzi G. Belletti N. (2007b). Effects of milk high pressure homogenization on biogenic amine accumulation during ripening of ovine and bovine Italian cheese. Food Chem. 104, 693–701. 10.1016/j.foodchem.2006.12.017

[B23] Lanciotti R. Patrignani F. Iucci L. Saracino P. Guerzoni M. E. (2007a). Potential of high pressure homogenization in the control and enhancement of proteolytic and fermentative activities of some Lactobacillus species. Food Chem. 102, 542–550. 10.1016/j.foodchem.2006.06.043

[B24] Lanciotti R. Vannini L. Patrignani F. Iucci L. Vallicelli M. Ndagijimana M.. (2006). Effect of high pressure homogenisation of milk on cheese yield and microbiology, lipolysis and proteolysis during ripening of Caciotta cheese. J. Dairy Res. 73, 216–226. 10.1017/S0022029905001640 16476182

[B25] Muramalla T. Aryana K. J. (2011). Some low homogenization pressures improve certain probiotic characteristics of yogurt culture bacteria and Lactobacillus acidophilus LA-K. J. Dairy Sci. 94, 3725–3738. 10.3168/jds.2010-3737 21787909

[B26] Patrignani F. Burns P. Serrazanetti D. Vinderola G. Reinheimer J. A. Lanciotti R.. (2009). Suitability of high pressure-homogenized milk for the production of probiotic fermented milk containing Lactobacillus paracasei and Lactobacillus acidophilus. J. Dairy Res. 76, 74–82. 10.1017/S0022029908003828 19121239

[B27] Patrignani F. Iucci L. Belletti N. Gardini F. Guerzoni M. E. Lanciotti R. (2008). Effects of sub-lethal concentrations of hexanal and 2-(E)-hexenal on membrane fatty acid composition and volatile compounds of Listeria monocytogenes, Staphylococcus aureus, Salmonella enteritidis and Escherichia coli. Int. J. Food Microbiol. 123, 1–8. 10.1016/j.ijfoodmicro.2007.09.009 18055050

[B28] Patrignani F. Iucci L. Lanciotti R. Vallicelli M. Mathara M. Holzapfel W. H.. (2007). Effect of high pressure homogenization, not fat milk solids and milkfat on the technological performances of a functional strain for the production of probiotic fermented milks. J. Dairy Sci. 90, 4513–4523. 10.3168/jds.2007-0373 17881672

[B29] Patrignani F. Tabanelli G. Siroli L. Gardini F. Lanciotti R. (2013). Combined effects of high pressure homogenization treatment and citral on microbiological quality of apricot juice. Int. J. Food Microbiol. 160, 273–281. 10.1016/j.ijfoodmicro.2012.10.021 23290235

[B30] Putignani L. Del Chierico F. Onorm M. Mancinelli L. Argentierim M. Bernaschi P.. (2011). MALDI-TOF mass spectrometry proteomic phenotyping of clinically relevant fungi. Mol. Biosyst. 7, 620–629. 10.1039/c0mb00138d 20967323

[B31] Russell N. J. Evans R. I. Ter Steeg P. F. Hellemons J. Verheul A. Abee T. (1995). Membranes as a target for stress adaptation. Int. J. Food Microbiol. 28, 255–261. 10.1016/0168-1605(95)00061-5 8750671

[B32] Somero G. N. (1992). Adaptations to hydrostatic pressure. Anim. Rev. Phys. 54, 557–577. 10.1146/annurev.physiol.54.1.557 1314046

[B33] Šedo O. Vávrová A. Vad'Urová M. Tvrzová L. Zdráhal Z. (2013). The influence of growth conditions on strain differentiation within the Lactobacillus acidophilus group using matrix-assisted laser desorption/ionization time-of-flight mass spectrometry profiling. Rapid Commun. Mass Spectrom. 27, 2729–2736. 10.1002/rcm.6741 24214857

[B34] Tabanelli G. Burns P. Patrignani F. Gardini F. Lanciotti R. Reinheimer J.. (2012). Effect of a non-lethal High Pressure Homogenization treatment on the in vivo response of probiotic lactobacilli. Food Microbiol. 32, 302–307. 10.1016/j.fm.2012.07.004 22986193

[B35] Tabanelli G. Patrignani F. Gardini F. Vinderola C. G. Reinheimer J. A. Grazia L.. (2014). Effect of a sub-lethal high pressure homogenization treatment on the fatty acid membrane composition of probiotic lactobacilli. Lett. Appl. Microbiol. 58, 109–117. 10.1111/lam.12164 24111720

[B36] Tabanelli G. Patrignani F. Vinderola G. C. Reinheimer J. A. Gardini F. Lanciotti R. (2013). Effect of sub-lethal high pressure homogenization treatments on in vitro functional and biological properties of lactic acid bacteria. Food Sci. Technol. 53, 580–586. 10.1016/j.lwt.2013.03.013

[B37] Vannini L. Lanciotti R. Baldi D. Guerzoni M. E. (2004). Interactions between high pressure homogenization and antimicrobial activity of lizozyme and lactoperoxidase. Int. J Food Microbiol. 94, 123–135. 10.1016/j.ijfoodmicro.2004.01.005 15193800

[B38] Vannini L. Patrignani F. Iucci L. Ndagijimana M. Lanciotti R.. (2008). Effect of a pre-treatment of milk with high pressure homogenization on yield as well as on microbiological, lipolytic and proteolytic patterns of “Pecorino” cheese. Int. J. Food Microbiol. 128, 329–335. 10.1016/j.ijfoodmicro.2008.09.018 18973961

[B39] Vinderola C. G. Prosello W. Ghiberto D. Reinheimer J. A. (2000). Viability of probiotic (Bifidobacterium, Lactobacillus acidophilus 512) and non probiotic microflora in Argentinean Fresco cheese. J. Dairy Sci. 83, 1905–1911. 11003217 10.3168/jds.s0022-0302(00)75065-x

